# Factors Associated with Success of Switching to Faricimab for Neovascular Age-Related Macular Degeneration Refractory to Intravitreal Aflibercept

**DOI:** 10.3390/life14040476

**Published:** 2024-04-04

**Authors:** Akira Machida, Akio Oishi, Junichiro Ikeda, Junko Kurihara, Ai Yoneda, Eiko Tsuiki, Yuki Hirata, Ryuya Murakami, Takashi Kitaoka

**Affiliations:** 1 Department of Ophthalmology and Visual Sciences, Graduate School of Biomedical Sciences, Nagasaki University, Sakamoto 1-7-1, Nagasaki 852-8102, Japan; a-machida@nagasaki-u.ac.jp (A.M.); piperh040826@gmail.com (J.I.); t-eiko@nagasaki-u.ac.jp (E.T.); haray@nagasaki-u.ac.jp (Y.H.); ryuya1130med@gmail.com (R.M.); tkitaoka@nagasaki-u.ac.jp (T.K.); 2 Department of Ophthalmology, The Japanese Red Cross Nagasaki Genbaku Hospital, Nagasaki 852-8104, Japan

**Keywords:** age-related macular degeneration (AMD), anti-vascular endothelial growth factor (VEGF) treatment, drug switch, intravitreal aflibercept (IVA), intravitreal faricimab (IVF), macular neovascularization (MNV), polypoidal choroidal vasculopathy (PCV), real-world date, refractory cases, treat-and-extend (TAE) regimen

## Abstract

We investigated the factors associated with the success of switching to faricimab for type 1 macular neovascularization (MNV) refractory to intravitreal aflibercept (IVA). This retrospective cohort study included patients with type 1 MNV who were switched to faricimab because they were refractory to IVA at two centers. The primary endpoint was a more than two-week extension of the treatment interval after 6 months. In addition, factors related to the success or failure of extension and visual and anatomical outcomes were assessed. The analysis included 43 eyes from 43 patients. Extended dosing intervals of >2 weeks were identified in 14 eyes (32.6%). A short dosing interval before switching, absence of polypoidal lesions, and thin central choroidal thickness before switching were identified as factors involved in successful extension. For patients with refractory type 1 MNV, switching to faricimab is a safe and potential option to extend existing dosing intervals.

## 1. Introduction

Age-related macular degeneration (AMD) is a leading cause of visual impairment, particularly in developed countries [1,2,3]. As shown in several large clinical trials [4,5,6], visual outcomes in patients with macular neovascularization (MNV) secondary to AMD have significantly improved since the introduction of anti-vascular endothelial growth factor (VEGF), which is currently the first-line treatment for neovascular age-related macular degeneration (nAMD). Although the need for long-term continuous treatment is a burden, the use of treat-and-extend regimens (TAE: shortened dosing intervals for active disease and extended dosing intervals for inactive disease) reduces patient visits and administrations compared to fixed dosing and maintains visual outcomes compared to PRN (dosing at exacerbations) [7,8].

However, a certain percentage of patients with nAMD is refractory to frequent anti-VEGF treatment over a long period. For example, in the VIEW1/VIEW2 trials, active exudation persisted in approximately 19.7% and 36.6% of the patients who received aflibercept treatment every 4 and 8 weeks for 1 year, respectively [9]. In a prospective multicenter study in Japan, the percentage of patients requiring monthly dosing after 2 years ranged from 16.5% to 20.6% [10], and 33.3% to −37.4% of patients required dosing every 8 weeks at 96 weeks [11]. Continuous treatment of these patients imposes a significant burden on both the patient and the physician, and there is great hope for alternative or additive treatments that can extend the dosing interval.

Faricimab is a bi-specific IgG1 antibody that targets VEGF-A and angiopoietin-2 (Ang-2), thereby suppressing conventional VEGF and Ang-2, which are involved in the regulation of vascular homeostasis, angiogenesis, and permeability [12,13]. Specifically, Ang-2 competes with Ang-1 to bind to Tie-2 receptors. Although Ang-1/Tie2 signaling promotes adhesion among endothelial cells and between endothelial cells and pericytes and reduces sensitivity to VEGF, excessive Ang-2 and subsequent blocking of this signal lead to pericyte loss, vascular leakage, and inflammation [14]. The process has been implicated in the pathogenesis of retinal vascular diseases, including nAMD. Several clinical trials, including those focused on nAMD, have demonstrated the efficacy of the anti-Ang-2 approach and have shown sustained clinical effects compared to conventional anti-VEGF agents [15].

In a clinical trial of patients with nAMD, faricimab exhibited efficacy, longer dosing intervals, and safety that were not inferior to those of aflibercept [16]. In real-world studies with patients who were resistant to aflibercept, switching to faricimab extended the dosing interval from 5.9 to 7.5 weeks or from 4.4 to 8.7 weeks, with success rates ranging from 29.1% to 40.8% [17,18]. Thus, switching to faricimab is a promising option for refractory cases. However, these studies did not identify predictive factors for switching success. Because faricimab is more expensive than other anti-VEGF agents, it is vital to identify patients who can benefit the most from the drug to ensure the appropriate allocation of resources.

In this study, we focused on type 1 MNV, subretinal pigment epithelium neovascularization, to minimize the confounding effect of lesion status and examined detailed background information to determine which patients would benefit from switching to faricimab.

## 2. Materials and Methods

### 2.1. Study Participants

This retrospective cohort study included patients who were continuously treated with intravitreal aflibercept (IVA) for nAMD at Nagasaki University Hospital and Nagasaki Genbaku Hospital according to the TAE regimen and who were switched to faricimab between August 2022 and March 2023. The inclusion criteria were as follows: (1) age over 50 years, (2) active type 1 MNV, (3) received IVA treatment with a TAE regimen, (4) an interval of 8 weeks or less between the last three doses of IVA, (5) a history of five or more consecutive IVA treatments, (6) switched to faricimab, and (7) at least 24 weeks of observation after switching. Only one eye from each patient was included in this study. The exclusion criteria were as follows: (1) axial length greater than 26.5 mm; (2) presence of inflammatory or hereditary diseases that may induce MNV; (3) previous treatment for MNV other than anti-VEGF injections, such as vitrectomy, photocoagulation, or steroid injection; (4) any other retinal or optic nerve disease; and (5) previous treatment for MNV at institutions other than the two centers of interest.

### 2.2. Intervention and Observation Procedure

We used a TAE regimen. Patients were initially treated with three monthly injections of aflibercept (loading phase), followed by additional injections. When switching to faricimab, the TAE regimen was continued without a loading phase starting at the pretreatment IVA interval. The interval between the next injection was extended by 1–2 weeks if there were no signs of exudation or worsening. Worsening was defined as an increase in intraretinal or subretinal fluid in the fovea as measured by optical coherence tomography (OCT) compared to the previous visit and the occurrence of subretinal hemorrhage based on fundus examination and fundus photography. If exudation or worsening was observed, the dosing interval for the next treatment was shortened by 1–2 weeks. The attending physician determined the interval between extension and shortening. Switching back to aflibercept was performed at the fifth dose of faricimab if the injection interval could not be maintained for >8 weeks, as faricimab should be administered at least eight weeks apart during the maintenance phase under the Japanese insurance system.

Patients underwent comprehensive examinations, including measurement of best-corrected visual acuity (BCVA), axial length (IOLMaster 500; Carl Zeiss Meditec, Dublin, CA, USA), fundus photography, spectral-domain optical coherence tomography (SD-OCT, Spectralis; Heidelberg Engineering, Heidelberg, Germany), fluorescein angiography, indocyanine green angiography, fundus autofluorescence imaging (HRA2; Heidelberg Engineering), and OCT angiography (Avanti; Optovue, Fremont, CA, USA) for diagnosis. BCVA was measured using Landolt C and converted to the logarithm of the minimum angle of resolution (logMAR) for statistical analysis; BCVA and SD-OCT measurements were performed at each visit. The SD-OCT images included 30° horizontal regular and enhanced depth scans through the fovea, and 15 raster scans covering a 20° × 15° oblong rectangle. Subretinal hemorrhage (SRH) was detected within 6 mm of the center based on fundus photographs and SD-OCT. Central choroidal thickness (CCT) was measured as the distance between the outer edge of Bruch’s membrane and the scleral interface using enhanced depth imaging scans. Pigment epithelium detachment (PED) height was defined as the distance between the outer edge of the retinal pigment epithelium and the inner edge of Bruch’s membrane, and the maximum height of the PED within 6 mm of the center was recorded. The presence of the ellipsoid zone (EZ) was evaluated on vertical and raster scans within 1 mm of the center. The disease type was determined using angiography, SD-OCT, OCT angiography, and fundus photography. Fundus angiography was performed before or immediately after the start of treatment unless the patient had an allergy to the contrast agent or other systemic risks. The nomenclature for nAMD is the same as that used in a previous study [19].

Two graders (J.I. and J.K.) blinded to the outcome performed the measurements and grading. The average of the measurements was used for the analysis, and discrepancies in grading were resolved by discussion.

### 2.3. Main Outcome Measure

**Figure 1:**
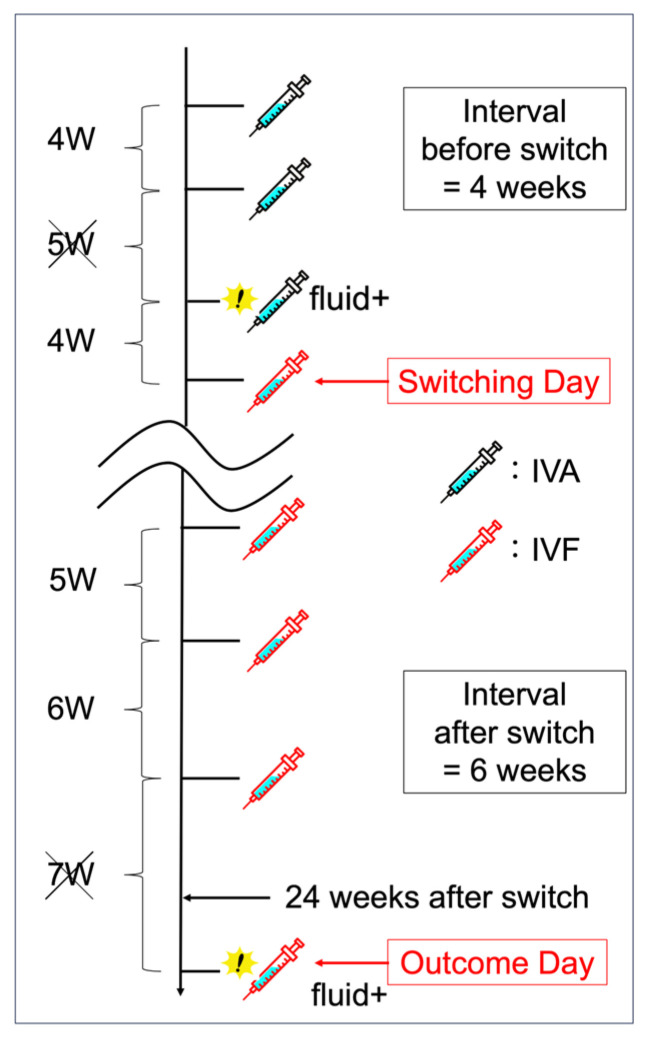
An example of how the pre- and post-switching dosing intervals is defined. The exclamation mark indicates that the disease activity appears. Of the three dosing intervals before switching, the longest interval without disease activity is 4 weeks, indicated by the cross sign. Of the last three dosing intervals from the outcome date, the longest interval without disease activity is 6 weeks. Since the dosing interval is extended from 4 to 6 weeks, this case constitutes a successful switching. IVA, intravitreal aflibercept; IVF, intravitreal faricimab.

The primary endpoint was successful switching, defined as at least a two-week extension of the treatment interval compared to the treatment interval before switching. Specifically, the pre-switch interval was defined as the longest interval without disease activity among the three aflibercept doses administered before the switch date. The post-switch interval was defined as the longest interval without disease activity between the last three faricimab doses before the outcome date. The outcome date was defined as the date of the 6-month follow-up visit or switchback (Figure 1).

### 2.4. Statistical Analysis

Values are presented as medians and interquartile ranges. All statistical analyses were performed using EZR [20], a modified version of the R commander for statistical functions commonly used in biostatistics. *p*-values were calculated using the Mann–Whitney U test, Fisher’s exact test, and the Cochran–Armitage trend test. Logistic regression analysis was performed using age, sex, presence of polypoidal lesions, history of cataract surgery and photodynamic therapy (PDT), number of previous anti-VEGF treatments, dosing interval before switching, baseline BCVA, central retinal thickness (CRT), CCT, maximum PED height, presence of exudative changes at switching, presence of SRH, and EZ status as independent factors and successful extension as the dependent factor.

## 3. Results

Based on these criteria, we enrolled 44 eyes of 44 patients with aflibercept-refractory nAMD during a specified period. One eye was excluded because of a decline in systemic diseases unrelated to the MNV treatment, and the final analysis included 43 eyes. Table 1 shows the background data of the 43 eyes. The study included 16 women and 27 men. The median age was 80.0 years (interquartile range: 73.0–84.5), 37.2% of the patients had a history of PDT, and the median number of previous anti-VEGF injections was 34.0 (20.5–52.5). Of the last three treatment intervals before switching, the median of the longest interval without exudative changes was 5.0 (4.0–6.0) weeks, with a logMAR acuity of 0.16 (0.05–0.30). Compared with those without polypoidal lesions (*n* = 14), the polypoidal choroidal vasculopathy (PCV) population (*n* = 29) was younger (*p* = 0.014), consisted of more males (*p* = 0.018), had a greater history of PDT (*p* = 0.006), and received fewer injections (*p* = 0.036). Before switching, the values for CRT, CCT, maximum PED height, presence of SRH, and EZ disruption were comparable regardless of the presence or absence of polypoid lesions.

**Figure 2:**
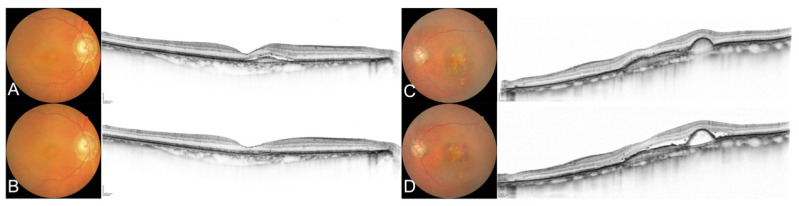
Color fundus photographs and optical coherence tomograms of representative cases of successful and failed switches to faricimab. (**A**) Case without polypoid lesions before switching (best-corrected visual acuity (BCVA) 20/25, dosing interval before switch was 5 weeks). (**B**) Images in case A at the first visit (5 weeks) after switching (BCVA 20/20). The dosing interval was eventually extended to 7 weeks at 6 months. (**C**) Case with polypoid lesions before switching (BCVA 20/40, dosing interval before switch was 6 weeks). (**D**) Images in case C after switching (BCVA 20/32, dosing interval 6 weeks). Early recurrence of subretinal fluid was observed after the switch, and the patient was eventually switched back to aflibercept.

Figure 2 shows the fundus images of a representative case of successful or failed switching. The successful switching group consisted of 14 eyes (32.6%) in which the treatment interval was extended by at least 2 weeks compared to the pre-switch interval. This group had fewer polypoidal lesions (*p* = 0.035), more previous cataract surgeries (*p* = 0.022), less previous PDT (*p* = 0.045), and shorter pre-switch dosing intervals (*p* = 0.035) (Table 2). Approximately half of the patients in the successful switching group showed no exudative changes at baseline, whereas those in the unsuccessful switching group showed more exudative changes (*p* = 0.040).

Logistic regression analysis identified three factors associated with successful switching: absence of polypoid lesions, CCT, and pre-switching interval. The odds ratios were 0.109 (95% confidence interval (CI), 0.019–0.642, *p* = 0.02) for polypoid lesions, 0.888 (95% CI, 0.789–0.999, *p* = 0.048) for every 10 μm increase in CCT, and 0.381 (95% CI, 0.168–0.864, *p* = 0.021) when the pre-switching dosing interval was extended by one week (Table 3).

The visual and morphological outcomes and treatment intervals over six months are shown in Table 4. The successfully extended group showed improvements in visual and overall morphological parameters. Visual acuity improved from 0.13 to 0.08 (logMAR), and CRT (277.0 to 248.0 μm), CCT (149.5 to 144.5 μm), and PED (178.5 to 152.5 μm) decreased at 6 months. Furthermore, the dosing interval was extended from five to eight weeks. In the group that failed to extend the dosing interval, CRT worsened (319.0 to 332.0 μm), but CCT (190.0 to 190.0 μm) and PED (198.0 to 182.0 μm) did not worsen, and visual acuity was maintained at 6 months (0.16 to 0.16). In the successful extension group, 78.6% of the patients experienced no or reduced exudative changes throughout the study period, whereas only 34.5% of the patients in the unsuccessful extension group experienced reduced exudative changes.

Five of the 43 eyes (11.6%) required faricimab injections for less than the 8-week interval. One eye was switched back to aflibercept on day 105 and four eyes were switched back to aflibercept at the 6-month outcome date. All switchback cases had polypoid lesions. They also had more previous PDT sessions and tended to have a higher median CRT; however, these differences were not statistically significant (Table 5). The logistic regression analysis revealed no significant background characteristics.

No adverse events such as retinal pigment epithelium tears, subretinal hemorrhage, intraocular inflammation, or cardiovascular events were observed in any patient after switching to faricimab.

## 4. Discussion

### 4.1. Factors Affecting Successful Switching

This is the first report to identify the factors associated with the success of switching to faricimab for aflibercept-refractory type 1 MNV. Switching from aflibercept to faricimab for refractory nAMD results in improved visual outcomes and longer dosing intervals in many cases [17,18,21,22,23]. However, the factors contributing to switch success have not been analyzed owing to the retrospective nature of the studies, short-term follow-up, and relatively small sample size of participants. The participants in the present study received treatment only at a designated facility for an extended period before switching. This enabled retrospective collection of information on the background characteristics of the target population. In addition, the results of the regression analysis were clarified by limiting the disease to type 1 to minimize confounding effects. Because faricimab is more expensive than other anti-VEGF drugs, it is important to identify patients who would benefit the most from this drug before switching.

Logistic regression analysis revealed that extension was more likely to be successful when the treatment interval before switching was short. One explanation for why faricimab was more effective in patients with shorter treatment intervals is that the anti-Ang-2 effect is more critical in populations that are highly resistant or tachyphylactic [24] to conventional aflibercept treatment. Elevated Ang-2 and subsequent inactivation of Tie-2 increase the sensitivity of endothelial cells to VEGF [25,26]. The anti-Ang-2 effect of faricimab may cancel hypersensitivity to VEGF in these patients. Switching to faricimab is recommended for patients with short dosing intervals.

The absence of polypoidal lesions and thinner CCT were also identified as background factors for successful switching. This is surprising because Ang-2 levels in the aqueous humor are higher in pachychoroid-associated MNV [27] than in drusen-associated MNV [28]. We assumed that faricimab improves elevated Ang-2 levels in patients with thick CCT and is more effective. We also assumed that patients with polypoidal lesions would show similar responses, because lesions are generally observed in patients with a thick choroid [29]. However, the results of this study are contradictory. A possible explanation for this is that patients with polypoidal lesions and thick choroids tended to undergo PDT before switching (15/29 patients with polypoidal lesions). Ang-2 inhibits Tie-2 signaling by competitively inhibiting Ang-1; Ang-2 inhibition promotes Ang-1-Tie-2 binding and stabilizes vessels via the activation of Tie-2 signaling [12]. This mechanism may be less advantageous for anomalous vessels that are occluded or degenerated by PDT. The findings of this study support switching to faricimab for non-PCV cases with thin CCT among refractory cases with a long treatment history.

### 4.2. Switching Success Rate

Similar reports in Japan have shown success rates of 29.1–40.8%. This is consistent with the prevalence of 32.6% reported in this study [17,18]. The finding that 30% of patients who are refractory to IVA can nearly double their treatment interval at six months is a great hope for patients who receive frequent conventional treatment. Additionally, the worsening of visual acuity in the population that failed to switch was minimal, and approximately 10% of the cases resulted in switchback owing to unacceptable switching. Switching is a relatively safe option in refractory cases.

### 4.3. Strengths and Limitations

The strength of this study is that the patients were treated using the same protocol and systematically examined, and the patient background, including age, disease type, anatomic parameters, previous treatment history (including PDT), and number of anti-VEGF treatments, was accurately collected. This is important for a multifaceted assessment of the success factors of switching.

The most significant limitations were the limited number of patients and selection bias associated with the retrospective design. PDT is often used in refractory cases with thick choroids, and there is a significant correlation among polypoid lesions, choroidal thickness, and PDT. The success rate of switching therapy may have been influenced by the fact that patients with a history of PDT had refractory AMD. In addition, loading injections were not administered during switching. Some of the patients with unsuccessful switching may have responded to monthly injections of faricimab. However, the optimal switching protocol requires further investigation. Because the study population did not include patients with prior treatment other than anti-VEGF therapy and PDT, the impact of other treatments, such as photocoagulation [30], transpupillary thermotherapy [31], micropulsed laser [32], and steroid injections [33], remains to be investigated. Nevertheless, we believe that these results are representative of typical clinical outcomes and can be applied to various populations.

## 5. Conclusions

In conclusion, this study demonstrated that switching to faricimab is effective in approximately 30% of the aflibercept-refractory population and that switching to faricimab is more successful in eyes without polypoidal lesions, thinner CCT, and short pre-switch intervals. Failure to switch was not strongly associated with the worsening of parameters, suggesting that switching is a safe option for treatment-resistant patients.

## Figures and Tables

**Table 1:** Clinical characteristics of patients with neovascular age-related macular degeneration who switched from aflibercept to faricimab and comparison of groups with and without polypoidal lesions.

	All	Without Polyp	With Polyp (PCV)	*p*-Value
	(*n* = 43)	(*n* = 14)	(*n* = 29)	
Age, years	80.0 (73.0–84.5)	83.5 (79.5–86.8)	75.0 (72.0–81.0)	0.014
Sex (female)	16 (37.2%)	9 (64.3%)	7 (24.1%)	0.018
Lens (IOL)	22 (51.2%)	9 (64.3%)	13 (44.8%)	0.332
History of PDT	16 (37.2%)	1 (7.1%)	15 (51.7%)	0.006
Total no. of previous injections	34.0 (20.5–52.5)	44.0 (34.0–61.8)	27.0 (13.0–52.0)	0.036
Interval before switch, weeks	5.0 (4.0–6.0)	5.0 (4.3–6.8)	5.0 (4.0–6.0)	0.904
BCVA, logMAR	0.16 (0.05–0.30)	0.10 (0.06–0.52)	0.15 (0.00–0.22)	0.412
Central retinal thickness, μm	289.0 (255.0–354.5)	299.5 (246.3–406.5)	289.0 (265.0–333.0)	0.959
Choroidal thickness, μm	161.0 (109.5–237.0)	159.0 (109.5–222.8)	161.0 (114.0–266.0)	0.907
Maximum PED, μm	193.0 (105.0–290.5)	176.0 (98.5–238.5)	201.0 (151.0–318.0)	0.294
Presence of SRH	3 (7.0%)	1 (7.1%)	2 (6.9%)	1.000
Disrupted foveal ellipsoid zone	21 (48.8%)	7 (50.0%)	14 (48.3%)	1.000

Continuous variables are presented as medians and quartile ranges. *p*-values were calculated using the Mann–Whitney U test and Fisher’s exact test. BCVA, best-corrected visual acuity; IOL, intraocular lens; logMAR, logarithm of the minimum angle of resolution; PCV, polypoidal choroidal vasculopathy; PDT, photodynamic therapy; PED, pigment epithelium detachment; SRH, subretinal hemorrhage.

**Table 2:** Comparison of baseline characteristics and changes in visual and morphological outcomes between patients with and without successful extension of treatment interval after switching to faricimab.

	Successfully Extended	Failed to Extend	*p*-Value
	(*n* = 14)	(*n* = 29)	
Age, years	82.5 (77.8–86.8)	77.0 (72.0–82.0)	0.094
Sex (female)	7 (50.0%)	9 (31.0%)	0.316
Subtype (PCV)	6 (42.9%)	23 (79.3%)	0.035
Lens (IOL)	11 (78.6%)	11 (37.9%)	0.022
History of PDT	2 (14.3%)	14 (48.3%)	0.045
Total no. of previous injections	36.5 (26.5–62.0)	34.0 (17.0–52.0)	0.300
Treatment interval, weeks	5.0 (4.0–5.0)	6.0 (5.0–6.0)	0.035
BCVA, logMAR	0.13 (0.10–0.28)	0.16 (0.00–0.30)	0.620
Central retinal thickness, μm	277.0 (246.3–310.5)	319.0 (265.0–387.0)	0.097
Choroidal thickness, μm	149.5 (115.3–179.0)	190.0 (102.0–286.0)	0.271
Maximum PED, μm	178.5 (98.5–238.3)	198.0 (151.0–318.0)	0.318
Presence of SRH	1 (7.1%)	2 (6.9%)	1.000
Disrupted foveal ellipsoid zone	7 (50.0%)	14 (48.3%)	1.000

Continuous variables are presented as medians and quartile ranges. *p*-values were calculated using the Mann–Whitney U test and Fisher’s exact test. BCVA, best-corrected visual acuity; IOL, intraocular lens; logMAR, logarithm of the minimum angle of resolution; PCV, polypoidal choroidal vasculopathy; PDT, photodynamic therapy; PED, pigment epithelium detachment; SRH, subretinal hemorrhage.

**Table 3:** Results of the logistic regression analysis with switching success as the dependent variable.

Covariates	β	Z-Value	Odd Ratio	95% CI	*p*-Value
With polypoidal lesions	−117.8	−0.001	0.109	0.019–0.642	0.014
Choroidal thickness, per 10 μm increase	−12.2	−0.001	0.888	0.789–0.999	0.048
Interval before switch, per week extension	−77.8	−0.002	0.381	0.168–0.864	0.021

β, regression coefficient; CI, confidence interval.

**Table 4:** Changes in visual and morphological outcomes and treatment intervals over 6 months in patients with and without successful retreatment after switching to faricimab.

	All	Successfully Extended	Failed to Extend	*p*-Value
	(*n* = 43)	(*n* = 14)	(*n* = 29)	
BCVA, logMAR	0.00 (−0.08–0.00)	−0.05 (−0.38–0.15)	0.00 (−0.10–0.30)	0.003
Central retinal thickness, μm	−2.0 (−36.0–25.0)	−29.0 (−51.5–−7.8)	13.0 (−12.0–32.0)	0.006
Choroidal thickness, μm	−1.0 (−13.5–8.0)	−5.0 (−18.8–5.3)	0.0 (−11.0–9.0)	0.190
Maximum PED, μm	−20.0 (−51.5–2.5)	−26.0 (−102.8–−13.3)	−16.0 (−33.0–9.0)	0.080
Exudative change				0.002
No fluid	4 (9.3%)	4 (28.6%)	0 (0.0%)	
Reduced	17 (39.5%)	7 (50.0%)	10 (34.5%)	
Worsened	22 (51.2%)	3 (21.4%)	19 (65.5%)	
Extended intervals, weeks	1.0 (0.0–3.0)	3.0 (3.0–4.8)	0.0 (0.0–1.0)	0.001

Continuous variables are presented as medians and quartile ranges. *p*-values were calculated using Mann–Whitney U, Fisher’s exact, and Cochran–Armitage tests. BCVA, best-corrected visual acuity; logMAR, logarithm of the minimum angle of resolution; PED, pigment epithelium detachment.

**Table 5:** Comparison of baseline characteristics and changes in visual and morphologic outcomes in patients who required switchback to aflibercept after switching to faricimab versus those who did not.

	Switched Back Cases	Others	*p*-Value
	(*n* = 5)	(*n* = 38)	
Age, years	77.0 (73.0–82.0)	80.0 (73.3–84.8)	0.622
Sex (female)	2 (40.0%)	14 (36.8%)	1.000
Subtype (PCV)	5 (100.0%)	24 (63.2%)	0.156
Lens (IOL)	3 (60.0%)	19 (50.0%)	1.000
History of PDT	4 (80.0%)	12 (31.6%)	0.056
Total no. of previous injections	27.0 (23.0–38.0)	34.5 (19.8–52.8)	0.733
Treatment interval, weeks	5.0 (5.0–5.0)	5.0 (4.0–6.0)	0.583
BCVA, logMAR	0.16 (−0.08–0.22)	0.13 (0.05–0.30)	0.746
Central retinal thickness, μm	340.0 (320.0–416.0)	283.5 (251.8–345.8)	0.099
Choroidal thickness, μm	127.0 (102.0–295.0)	161.0 (114.0–226.0)	0.880
Maximum PED, μm	202.0 (179.0–318.0)	190.5 (103.8–263.3)	0.405
Presence of SRH	0 (0.0%)	3 (7.9%)	1.000
Disrupted foveal ellipsoid zone	2 (40.0%)	19 (50.0%)	1.000

Continuous variables are presented as medians and quartile ranges. *p*-values were calculated using the Mann–Whitney U test and Fisher’s exact test. BCVA, best-corrected visual acuity; IOL, intraocular lens; logMAR, logarithm of the minimum angle of resolution; PCV, polypoidal choroidal vasculopathy; PDT, photodynamic therapy; PED, pigment epithelium detachment; SRH, subretinal hemorrhage.

## Data Availability

Access to data and data analysis: A. Machida had full access to all data in the study and took responsibility for the integrity of the data and the accuracy of data analysis. The data supporting the findings of this study are available upon request from the corresponding author. The data are not publicly available because of restrictions that include information that could compromise the privacy of the research participants.
